# Association between monosodium glutamate consumption with changes in gut microbiota and related metabolic dysbiosis—A systematic review

**DOI:** 10.1002/fsn3.4198

**Published:** 2024-04-29

**Authors:** Hossein Ahangari, Behnam Bahramian, Arezou Khezerlou, Milad Tavassoli, Narges Kiani‐Salmi, Vahideh Tarhriz, Ali Ehsani

**Affiliations:** ^1^ Student Research Committee Tabriz University of Medical Sciences Tabriz Iran; ^2^ Department of Food Science and Technology, Faculty of Nutrition and Food Sciences Tabriz University of Medical Sciences Tabriz Iran; ^3^ Cardiovascular Center of Excellence Louisiana State University Health Sciences Center New Orleans Louisiana USA; ^4^ Nutrition Research Center Tabriz University of Medical Sciences Tabriz Iran

**Keywords:** dysbiosis, food additive, gut microbiota, monosodium glutamate

## Abstract

Monosodium glutamate (MSG) is used as a common food additive in some foods. However, based on our search and knowledge, no comprehensive study discussed the effect of MSG on the human gut microbiome. In this study, the effects of MSG on the gut microbiome, liver, and kidney were performed. Data were collected from databases including PubMed, Scopus, Web of Science, and ScienceDirect using the search strategy and keywords. Finally, 14 eligible studies were selected for systematic review. This study provides a new perspective on the effects of MSG on the gut flora, shedding light on the potential relationship between MSG intake and human health.

## Graphical abstract

MSG consumption can increase the hazard of cardiovascular illness, and in addition, disrupts the intestinal microbiome and intestinal function. In general, the results of this study provide a new perspective and basis for future studies on the effects and safety of MSG on human health.

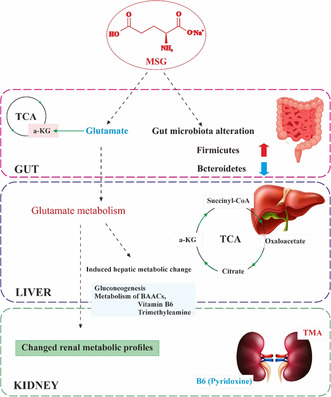

## 1 INTRODUCTION

For the first time in 1908, Professor Kikona Ikeda, introduced the MSG to the world as the taste of umami (Bayram et al., 2023). MSG is one of the most common flavor enhancers in food, which increases the intensity of the taste and improves palatability (Shosha et al., 2023). The sodium salt of glutamic acid is called MSG (C_15_H_8_NO_4_Na), which consists of glutamic acid, sodium, and water (Onyesife et al., 2023). Glutamic acid occurring naturally in food does not pose any health problems, while the formation of glutamic acid during industrial processes can have toxic effects on living cells (Chakraborty, 2019). MSG is typically considered as a safe food additive by the FDA, EFSA (European Food Safety Authority), and the Joint FAO/WHO Committee on Food Additives (JECFA). These organizations have evaluated MSG and determined it to be safe for consumption within specified limits.

According to the FAO and WHO guidelines, the acceptable daily intake (ADI) of MSG is 120 mg/kg/bw/day (Xu et al., 2022). However, re‐evaluations in 2017, EFSA proposed the ADI of 30 mg/kg/bw/day for using glutamate and its related salts as a food additive (Akshata et al., n.d.; Mennella et al., 2023). It is worth noting that laboratory animal studies have shown adverse effects of chronic consumption of MSG in body organs such as the liver, brain, pancreas, kidney, and testis, along with increasing the prevalence of diseases including hypertension, obesity, asthma, exacerbation, headaches, detrimental, neurotoxicity, and effects on the reproductive organs (Hajihasani et al., 2020). Ingesting MSG can lead to oxidative stress, which can exacerbate the chronic effects mentioned earlier (Kamal et al., 2023). Foods high in protein, such as cheese, meat, eggs, fish, and certain vegetables like tomatoes, mushrooms, and green beans, as well as fermented foods like fermented bean paste and soy sauce, are the primary sources of glutamate (Hamad, 2022; Xu et al., 2022).

The human intestine is composed of 100 trillion microbial cells collectively known as gut microbiome or microbiota (Wang et al., 2023). Various factors including diet, metabolism, immunity, mental and physical stress, antibiotics, ethnicity, gender, age, intestinal milieu, and geographic location influence the gut microbiota (Lu et al., 2022; Pongking et al., 2020). Recent studies have shown that the gut microbiota have a significant impact on various physiological functions, including mucosal immune homeostasis, immuno‐modulation, supporting intestinal permeability, and the integrity of host epithelium, as well as inhibiting the colonization of pathogens. Additionally, gut microbiota plays a crucial role in energy metabolism, fat storage, vitamin synthesis, fermentation of monosaccharides, degradation of polysaccharides, reduction of cholesterol, and metabolism of drugs or xenobiotic (Roshanravan et al., 2023). The gut microbiota can be manipulated using pro‐ and prebiotics to control metabolic syndromes. The application of certain probiotic species together with different prebiotics has demonstrated promising results in improving lipid profiles, glycemic control, and inflammatory markers in human subjects (Hadi et al., 2021). Recent studies demonstrated that dairy or nondairy fermented foods are great sources of pro‐ and prebiotics, or as nutrition supplements, which can be useful in certain medical disorders, including gastroenteritis, and respiratory tract infection (Tavakoly et al., 2021).

Recent studies have shown that long‐term consumption of MSG has a negative impact on the population of beneficial bacterial species such as *Lactobacillus* sp. and other probiotics in the gut, while the population of certain pathogens such as *Escherichia* and *Bacteroides* are increased, which means the double side effects of the MSG on intestinal flora. MSG also affects other related species and genera, especially *Megamonas*, *Faecalibacterium*, and *Blautia* species, which are decreased, while the *Collinsella* species is increased (Peng et al., 2018). Exopeptidase enzyme naturally occurs in human intestine and is responsible for proteins break down. MSG is generally found in hydrolyzed vegetable protein, which activates orosensory receptors and improves the taste of food. However, excessive consumption of MSG can affect the appetite center, leading to obesity (Zanfirescu et al., 2019). According to previous research, both humans and experimental animals may receive harmful effects from relatively low doses of MSG (0.6 and 1.6 mg/g body weight for 2 weeks or 100–500 mg/kg body weight for 3 weeks). Conducting a comprehensive experimental study to ascertain the toxicity, long‐term intake of MSG in individuals is challenging due to various factors such as moral concerns, and dietary guidelines (Umukoro et al., 2015; Zanfirescu et al., 2019). However, quantifying excitotoxic neurotransmission in mammals may be effectively achieved using rodent models. Consequently, rodent models are the subject of the vast majority of review publications. Researchers have shown that MSG‐induced dyslipidemia modifies the LDL/HDL ratio in obese subjects without causing a hyperphagic response, which results in heightened insulin levels, fibrosis, and steatosis in rats (Fujimoto et al., 2014). Furthermore, some studies showed that MSG induced hepatocellular injury by changing liver metabolism and raising the levels of alanine aminotransferase (ALAT), aspartate aminotransferase (ASAT), and gamma‐glutamyl transferase (Manal Said & Nawal, 2012). MSG has been associated with hepatocellular apoptosis through various theories, including the presence of structural anomaly in the mitochondria and endoplasmic reticulum with karyopyknosis that is undergoing apoptosis, as well as the upregulation of apoptotic mediator proteins like p53 and ki‐67 (Osman & Daghestani, 2012).

According to previous studies, there is correlation between MSG consumption and gut microbiota, as well as metabolic dysbiosis that may be related to it. Therefore, the purpose of this study is to conduct a systematic review of the association between MSG intake and modifications in intestinal flora, as well as any associated metabolic dysbiosis.

## 2 METHOD

### 2.1 Search strategies

**FIGURE 1:**
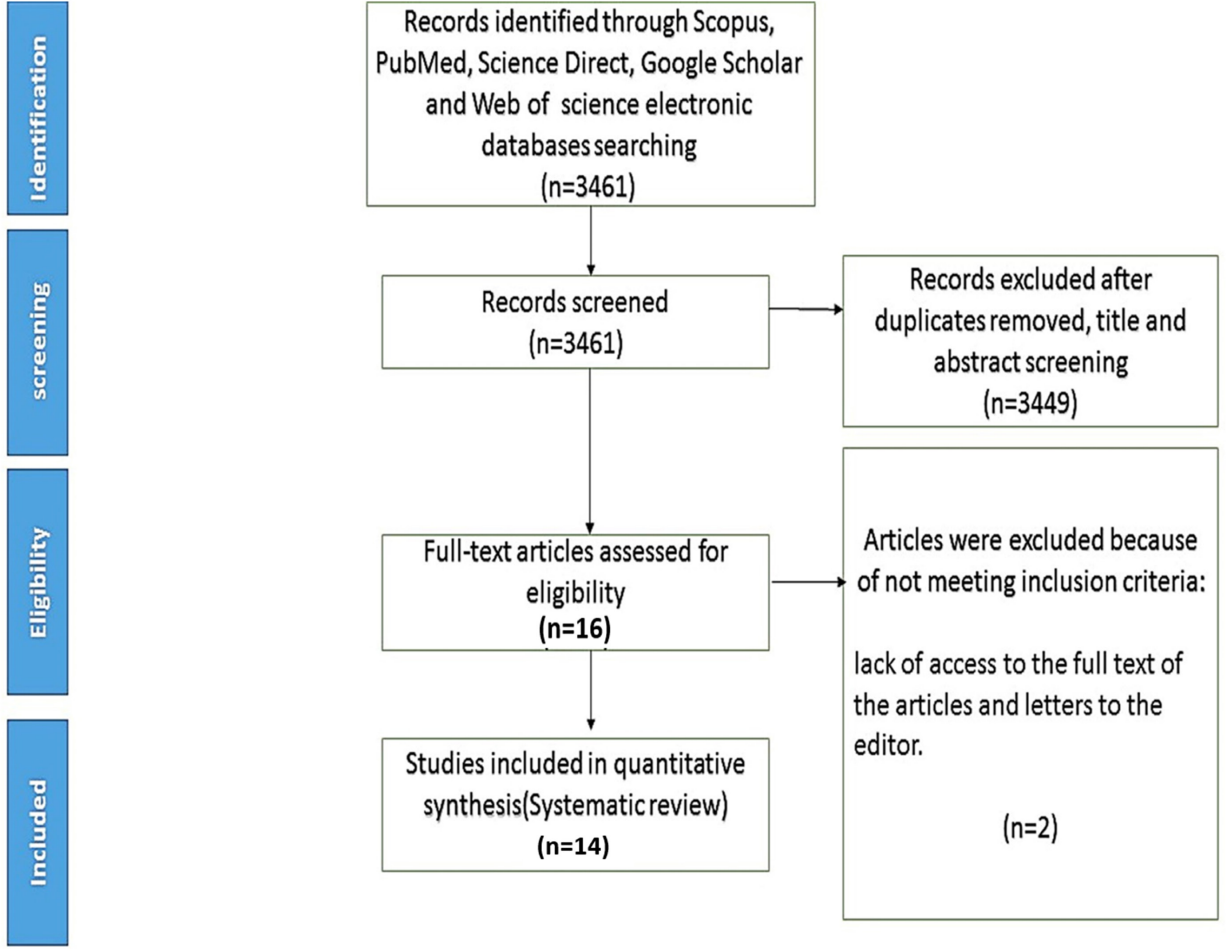
The screening method of included studies from scientific databases.

A systematic literature search have been conducted through databases including Scopus, ScienceDirect, Web of Science, and PubMed for articles published during 2000–2024 February (Figure 1). In addition, the reference list of related articles was checked to find more articles. The search strategy was to combine searches of monosodium glutamate, and gut microbiota with the operators “OR” and/or “AND.” A total 14 articles were chosen after conducting eligibility analysis and cross‐referencing, in accordance with the guidelines of the systematic review‐PRISMA (Transparent reporting of systematic reviews and meta‐analyses, http://www.prisma‐statement.org). A complete electronic search strategy for PubMed is provided in Table S1.

### 2.2 Selection criteria and quality assessment

Two authors (H.A and B. B) independently performed the search and data extraction process. They reviewed the abstracts, titles, and full texts of articles that met the inclusion criteria, and again, the databases were reexamined by the authors (A.K and M.T) to resolve any discrepancies.

The inclusion criteria for this article were access to the full text of the articles, a review of MSG safety studies, and a review of studies on the effects of MSG on gut microbiota. The exclusion criteria were as follows: Letter to the editor and lack of access to the full text of the articles.

## 3 RESULTS AND DISCUSSION

### 3.1 Effect of monosodium glutamate on body function

There have been few studies conducted on safety of the MSG. One such study was conducted by Xu et al. (2022), who investigated the safety of MSG based on function of gut flora in mice, and showed that the levels of angiotensin II, trimethylamine oxide (TMAO), and C‐reactive protein did not increase in the supplement of 30 mg/kg compared to the control group of mice. However, in the 300 mg/kg supplement, only the angiotensin II biomarker increased in comparison to the control sample. In addition, in the 1500 mg/kg supplement, all three biomarkers were found to be increased. C‐reactive protein is a sensitive inflammatory biomarker that is generally used to specify disease and inflammatory activity (Escadafal et al., 2020). TMAO is produced from trimethylamine metabolism by gut microbiota and eliminated from the body by the kidney. Previous studies reported that TMAO can interfere with the reverse transport of cholesterol, facilitating the release of inflammatory cytokines and increase the risk of developing cardiovascular disease (CVD) (Seldin et al., 2016). Another study showed that consumption of MSG by male rats increased the serum level of TMAO and decreased TMAO excretion in urine (Kyaw et al., 2022). Studies have shown that elevated plasma TMAO concentration leads to fat deposition in blood vessels, which promotes the development of atherosclerosis and increases the risk of myocardial infarction and stroke (Randrianarisoa et al., 2016; Stremmel et al., 2017; Tang et al., 2015; Velasquez et al., 2016; Wang et al., 2011). Xu et al. (2022) reported that a dose of 30 mg/kg of MSG led to the growth of intestinal villi, which could be attributed to the metabolism of MSG by epithelial cells. In addition, the results showed that 1500 mg/kg of MSG caused intestinal permeability to be disturbed, and albumin leaked as a result. This damage was linked to the disruption of osmotic pressure in the intestine due to the excessive accumulation of sodium ions, which resulted in harm to the bowel barrier structure.

In a study by Nakadate et al., 2016, the pathological changes of small intestinal epithelial cells in MSG‐induced obesity have been determined. The obese mice did not show any changes in their macroscopic anatomy; however, a closer look using light microscopy revealed that their small intestine had thinned and elongated villi. In a similar study, rats that were given MSG at 4, 8, and 12 weeks of age showed that the length of their intestinal villi gradually increased as they aged. (Hamaoka & Kusunoki, 1986). The hyperplasia in small intestine is possibly due to the acceleration of absorptive function. In another study by Nemeroff et al. (1977), consumption of MSG from 1 month of age led to decreased thyroid hormone levels and less spontaneous activity in obese mice compared to the control group (Nemeroff et al., 1978). On the other hand, the authors showed that in the stretched villi of intestinal mucosa, might induce the nutrients absorption and obesity. According to the results of the study by Nakadate et al. (2016), MSG‐treated obese mice showed decreased proliferation of the Golgi apparatus in the epithelium of the small intestine and the amount of rough endoplasmic reticulum (rER). The rER has numerous critical functions, including the protein transportation in vesicles to the Golgi apparatus and the folding of proteins in cisternae; therefore, these functions could be affected by changes in the rER of intestinal mucosa cells. Pongking et al. (2020) have studied the effect of MSG consumption and high fat and fructose (HFF) diet on the composition of gut microbiota with co‐observation of urine metabolite alteration and kidney injury in hamsters. The 20 mg/mL dose of MSG was applied based on an average intake of MSG in humans in the range of 0.4–14 mg/day (Insawang et al., 2012). In a study, it was reported that the MSG decreased the gene expression of tight junction proteins (Occludin) in the colon (Z. Feng, Li, Wu, Tao, et al., 2015).

Based on these findings, the study showed that enzyme activity in the small intestine plays a crucial role in regulating energy balance in MSG‐induced obese rats. This finding suggests that MSG overconsumption can affect various factors, including enzymatic activity, leading to changes in gut microbiota (Mozes et al., 2004a). As previously mentioned, both genetic and physiological factors can affect the composition of gut microbiota. Alongside these, dietary fat, temperature, and the use of antibiotics can also have an impact. However, it is not yet clear how MSG affects these factors, and subsequently, how it may alter the gut microbiota (Z. Feng, Li, Wu, Tao, et al., 2015).On the other hand, the ingestion of high concentrations of glutamate in the form of monosodium salts can lead to neurotoxicity through the over‐activation of glutamatergic receptors (Choudhary et al., 1996). This point highlights the neurodegenerative effect of MSG on the brain, particularly in immature animals, and causes damage in specific regions of the brain where there is no blood–brain barrier, including the hypothalamus' arcuate nucleus (Hinoi et al., 2004). Moreover, high levels of MSG intake can cause kidney injury by increasing the production of reactive oxygen species (ROS), which in turn triggers interstitial fibrosis in renal tubules and development of the chronic kidney disease (CKD) (Cerdá et al., 2016).

Ortiz et al. (2006) conducted a study to investigate the effects of MSG injection on the enzyme level, lipid peroxidation, and morphological changes in the kidney and liver of rats. The study found that MSG injection increased the activity of ALAT and ASAT enzymes. Injection of 4 mg/g body weight of MSG caused an increase in the levels of malondialdehyde (MDA) and 4‐hydroxyalkenals as a response to liver and kidney damage. The kidneys and livers of rats injected with MSG were pale in color with clear symptoms of edema, congestion, and loss of hepatic borders. When MSG is converted to glutamine in hepatic cells, the cells try to repair part of the damage using enzymes produced by the smooth endoplasmic reticulum. However, the liver is unable to remove the excessive levels of glutamine, leading to turbid swelling, vesicular degeneration, and ultimately necrosis (Ortiz et al., 2006).

In a study performed by Sharma et al. (2013), it was found that Wistar rats treated with 2 mg/g of body weight of MSG for 9 months developed lithiasis. The rats fed with MSG showed the presence of numerous kidney stones with smooth surfaces in the calyx and pelvis, along with typical features of hydronephrosis, as observed by gross anatomical inspection of the kidneys. The MSG‐treated rats also showed significantly higher urine pH and higher serum creatinine levels. This study was the first to report that chronic ingestion of dietary MSG can cause urolithiasis and obstructive nephropathy in adult rats by increasing the urine's alkalinity. The main mechanism responsible for this is not yet known; however, it is assumed that the catabolic products of glutamate in kidney cells are converted into bicarbonate anion, which is then absorbed for excretion by the kidney. Oral consumption of MSG increases urinary pH, and a renal compensation mechanism neutralizes this alkalinity by elevating the excretion of organic anions such as citrate during the alkali load (Sharma et al., 2013).

Paul et al. (2012) conducted a study to investigate the effect of α‐tocopherol in protecting against oxidative stress in Wistar rats orally treated with MSG at a dose of 4 g/kg/day. The results showed that chronic intake of MSG led to a significant increase in the levels of serum urea, creatinine, and uric acid. There was also an increase in lipid peroxidation markers, including MDA and conjugated dienes (CD), in renal tissues. Moreover, the activity of antioxidant enzymes, such as glutathione peroxidase (GPx), catalase (CAT), superoxide dismutase (SOD), and glutathione transferase (GST), was significantly decreased after MSG administration. Histopathological examination of the kidney in the MSG‐fed group revealed cloudy swelling of the tubules, glomerular and vascular congestion, and microhemorrhages in stromal areas. These findings are consistent with other studies that report the nephrotoxicity of MSG (Paul et al., 2012).

A study conducted by Insawang et al. (2012) found a link between the daily consumption of MSG and the risk of metabolic syndromes in a rural area of Thailand. In this study, 324 families with 487 participants were involved, and they were given 250 g of edible MSG to be consumed over 10 days as a flavor enhancer in their food preparation. The results indicated that MSG intake led to an increase in the prevalence of elevated blood pressure, hyperglycemia, and enhanced waist circumference. However, these differences did not show any statistical significance. After adjusting the data for potential confounding factors, the results pointed out that MSG consumption was independently linked to having metabolic syndrome. Meanwhile, increasing the ingestion of MSG slightly increased the odds of metabolic syndrome and overweight. Furthermore, the study found a significant trend in increasing insulin levels and prevalence of insulin resistance across groups receiving MSG. However, glucose homeostasis did not show any significant changes. The findings of the animal model studies were consistent with those of this study. MSG consumption was found to increase the rate of lipogenesis and enhance the shifting of dietary glucose toward lipid synthesis by activating the gene expression of enzymes involved in lipid biosynthesis (Insawang et al., 2012).

### 3.2 Microbial and metabolites changes

As reported by Kyaw et al. (2022), the gut microbiota causes significant changes in *Verrucomicrobia* and *Firmicutes* by modification in TMAO production. On the other hand, no significant changes have been reported in trimethylamine (TMA)‐producing bacteria within the *Streptococcus*, *Clostridium*, *Enterococcus*, *Desulfitobacterium*, *Staphylococcus, Haloanearobacter*, which are from phylum *Firmicutes* and *Proteobacteria* (Romano et al., 2015). As stated by the authors, effective factors in the modification of gut microbiota were genetics, the pH of the lumen, and age. In addition, researchers have proven that MSG suppresses the number of Verrucomicrobia spp. as beneficial bacteria in the gut (Peng et al., 2018). Further, *Akkermansia muciniphila*, a subphylum of Verrucomicrobia, was suppressed, related to the TMAO level (Griffin et al., 2019) and precisely implicated in the mucus thickness, gut barrier, and even immune responses (Ottman et al., 2017), with valuable clinical associations (Zhang et al., 2019). The reverse effect of MSG on *Lactobacillus intestinalis* in comparison with *A. muciniphila* may be a consequence of the compensation effects to homeostasis sustaining in the host since both bacterial species apply the functional roles for the protection of the gut barrier (Lim et al., 2021). The researchers found that MSG intake was also associated with water intake and urine output (Elliot et al., 1996). It is possible to evaluate the level of MSG in animals by measuring their urine output and water intake. Previous studies have shown that MSG increases the serum level of TMAO, which is produced by the gut microbiota through the metabolism of TMA. Additionally, MSG has been found to decrease the renal excretion of TMAO (Zeisel & Warrier, 2017).

Mozes et al. (2004a) investigated the effect of MSG on alkaline phosphatase levels, which is an enzyme involved in the absorption of nutrients, especially fat, and the transportation of long‐chain fatty acids. Yeh et al. (1994) reported that the level of alkaline phosphatase mRNA in the small gut of lactating rats increased over time from Day 12 to Day 24, and on Day 14 led to a decrease in the level of alkaline phosphatase in the whole membrane of the small intestine compared to the normally fed rats. However, the results of Mozes et al. (2004b) demonstrated the increased activity of alkaline phosphatase in 40‐day‐old obese control rats and MSG‐treated rats. Both groups exhibited normophagia, indicating changes as a result of postnatal overnutrition. However, MSG‐treated rats showed significantly higher alkaline phosphatase activity suggesting that MSG may cause persistent changes in the small bowel's function. The consumption of MSG can affect these factors and lead to changes in the population of gut microbiota.

**FIGURE 2:**
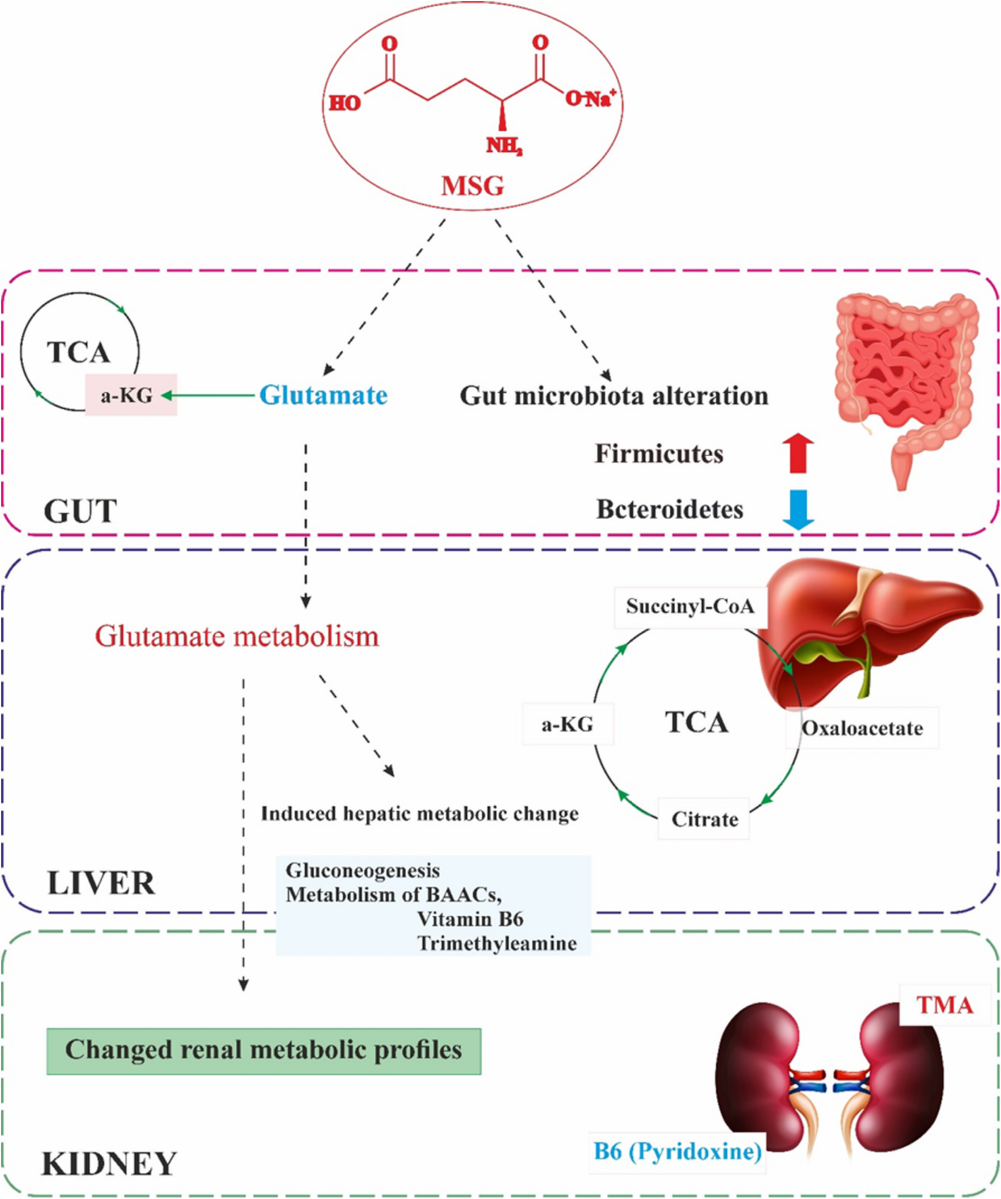
Mechanism of MSG and derived metabolites on gut microbiota modulations, kidney, and liver injury.

Researchers have shown that a high‐salt diet can have detrimental effects on the function and structure of the intestinal microbiota, including *Lactobacillus* (Miranda et al., 2018). In this way, Peng et al. (2018) have explored the effects of MSG on composition of the gut microbiota through high‐throughput sequencing. The findings revealed that gut microbiota diversity was similar at various MSG consumption phases. In addition, the following principal coordinate analysis showed there was no substantial difference in the microbial composition before and after MSG exposure, indicating that the MSG effect was insignificant compared to host genotypes. Overall, *Bacteroidetes* and *Firmicutes* were described as the two main phyla in all the samples, consistent with previous studies that have identified these phyla as the most abundant in the human gut. (Claesson et al., 2009). The authors reported no significant change in intestinal bacterial population due to MSG consumption; however, a particular variation trend of specific genera was stated. A study showed that, the abundance of *Faecalibacterium*, *Blautia*, and *Megamonas* decreased slightly, but *Collinsella* increased in MSG consumption (Maslowski et al., 2009). *Blautia* is considered as one of the useful intestinal bacteria as a short chain fatty acids producer (SCFAs) (Zhang et al., 2015). In contrast, Sun et al. (2018) reported a suggestive association between *Megamonas* and systemic inflammatory cytokines as well as endotoxin, both of which are known to be increased in Budd–Chiari syndrome, and their levels experienced a slight decline throughout the experiment. Figure 2 presented the mechanism of MSG and derived metabolites on gut microbiota modulations, kidney, and liver injury.

Xu et al. (2022) reported that high MSG consumption might affect the survival of some bacterial species due to their different salt tolerance. In this study, various doses of MSG had no significant effects on *Bacteroidetes* and *Firmicutes* populations of rat's intestine. This indicated that MSG might have minor effects on the gut microbiome population. Additionally, multiple studies have demonstrated that consumption of MSG alone in pigs did not significantly affect the *Bacteroidetes* and *Firmicutes* populations (Feng, Li, Wu, Xiao, et al., 2015). Remarkably, MSG promoted the growth of *Patescibacteria*, which is widely found in soil, seawater, and the animal's digestive tracts. Changes in several *Lachnospiraceae* NK4A136 groups, *Roseburia* and *Blautia*, have been detected at the genus level, which are SCFAs‐producing genera (T. Liu, Guo, et al., 2020). SCFAs play significant roles in ameliorating obesity, hypertension, and dyslipidemia (Pongking et al., 2020), and previous studies indicated that the *Methanobrevibacter* promotes polysaccharides fermentation by H_2_ removal, and increases the levels of SCFAs in the colon and adipose tissue (Jiang et al., 2017).

Moreover, the population of beneficial bacterial genera *Lactobacillus* (MN326537) and *Allobaculum* (MN326542) were decreased in hamsters with MSG and HFF diets (Jakobsson et al., 2015). These findings were evidenced by Liu, Chen, et al. (2020) in a study on the effects of MSG on the composition of human gut microbes and production of SCFAs. In this survey, the in vitro fermentation of gut microbiota using MSG as substrate was studied and the SCFAs and γ‐aminobutyric acid (GABA) content were determined. The results showed that the total level of SCFAs in the MSG fermentation was significantly higher than in the control models, with a 26‐fold increase in butyric acid content.

Moreover, excessive intake of MSG enhances the abundance of *Bacteroidetes* and *Alistipes*. *Alistipes* stimulate the inflammatory reactions of host and are abundant in type 2 diabetic patients (Wan et al., 2019). Findings implied that MSG intake might improve intestinal flora by enhancing amino acid and energy supply. High doses can cause dysbiosis and increase illness‐associated microbiota. According to previous studies, the imbalance of *Firmicutes* and *Bacteroidetes* is related to obesity (Macfarlane & Macfarlane, 1997) and a higher *Bacteroidetes*/*Firmicutes* is proved ratio in gastrointestinal tract of obese animal models and humans (Kalliomäki et al., 2008). The reviewed studies indicated that MSG has effects on both beneficial or pathogen bacterial groups, as indicated by Liu, Chen, et al. (2020), by in vitro fermentation of MSG, the relative abundance of *Proteobacteria* and *Bacteroidetes* phyla increased, while the *Actinobacteria* and *Firmicutes* phyla decreased. Additionally, the genera *Escherichia*, *Shigella* and *Bacteroides* experienced an increase in population.

In a study by Feng, Li, Wu, Xiao, et al., 2015, the consumption of MSG in a high‐fat diet modified the intestinal microbiota composition by increasing the *Bacteroidetes*/*Firmicutes* ratio within a small range. Contrary to reported studies, the ratio of *Firmicutes*/*Bacteroidetes* was reduced in the jejunum, suggesting that modification in composition of gut microbiota might be dependent on anatomical compartments. However, no noticeable effect on the microbiota composition was observed when MSG was given alone. At the same time, the individual supplementation of either MSG or fat diet did not affect the *Bacteroidetes*/*Firmicutes* ratio in the colon. However, when the two nutrients were included simultaneously, this ratio was increased. According to this study, dietary fat increased the proportion of colonic *Methanobrevibacter smithii*, a hydrogen‐consuming methanogen (Eckburg et al., 2005), while MSG exhibited the converse effect. However, the obtained results revealed no association between the relative number of this bacterial species and dietary fat consumption. The *M. smithii* can stimulate *Bacteroides thetaiotaomicron* to produce formic acid by decomposing the fructose (Samuel et al., 2007), which can act in synergistic effect for energy harvesting from polysaccharides (Samuel & Gordon, 2006). Even though *Bacteroides thetaiotaomicron* was not detected in this study that suggests a high‐fat diet, MSG can affect the colonization of *M. smithii* and amount of the polysaccharide broken down in the intestines. Similarly, dietary fat increases the ratio of *Peptostreptococcus* products, which can degrade lignin (Clavel et al., 2006). At the same time, its relative quantity in the colon was decreased, signifying that the effect of dietary adjustments may be dissimilar based on the segment of the intestine. In a study, MSG was found to decrease the presence of *Peptostreptococcus* products in both the jejunum and colon, indicating the complex interactions of these dietary compounds on the intestinal microbiome. The results also showed that the species most impacted by MSG and dietary fat was *Fusobacterium prausnitzii*. It has been reported that more *Faecalibacterium prausnitzii* were found in the gut of obese persons than in their lean counterparts (Balamurugan et al., 2010). Dietary supplementation with both MSG and high fat enhances the percentage of these two bacterial species by three to sixfold. Besides, the addition of MSG has shown no effect on *Prevotella*, which has a great capability in fermentation and hydrolysis of dietary fibers to form propionic acid and acetic acid. In conclusion, this survey revealed that both MSG and dietary fat intensified intestinal microbiota diversity, which can be considered paradoxical to some previously published studies. The results indicated that the combination of HFF and MSG diets led to modifications of the gut microbiota, damage to kidney tissues, a decrease of urine TMAO and indoxyl sulfate, and intensification of p‐cresol sulfate production level. The sequencing of prokaryotic 16S rRNA sequences in V3‐V4 regions showed that the HFF + MSG diet could alter the gut microbiota composition. These findings aligned with Han et al. (2015) by the high ratio of *Bacteroidetes*/*Firmicutes* in the HFF + MSG diet group. The diversity tests revealed that changes of gut microbiota composition in MSG‐treated hamsters, were insignificant, which is in line with a former report in humans. MSG diet has led to proliferation of *Citrobacter* (MN326558), *Ruminococcus*‐1 (MN326532), and *Roseburia* (MN326554), whereas the increase of *Roseburia* after MSG consumption has been reported again (Peng et al., 2018). *Roseburia* is a class of butyrate‐producing anaerobic gut bacteria that is negatively dependent to CKD progression, and the increase of *Roseburia* (MN326554) population might be due to intake of MSG since the glutamate can be converted to butyrate (Jiang et al., 2016). The *Citrobacter* genus produces the tyrosine phenol‐lyase enzyme, which converts L‐tyrosine to 4‐hydroxyphenylpyruvate and metabolizes into p‐cresol sulfate, which induce toxicity (Kikuchi et al., 2019). It was previously reported that the MSG + HFF dietary supplementation intensifies the *Fusobacterium prausnitzii*, *Clostridium coccoides*, *F. prausnitzii*, *Peptostreptococcus*, *Roseburia*, and *Prevotella* in the cecum; however, it decreases the *Clostridium leptum* and *Bacteroides thetaiotaomicron* the subgroup (Feng, Li, Wu, Xiao, et al., 2015). The levels of beneficial bacterial genera, including *Allobaculum* (MN326542) and *Lactobacillus* (MN326537) were lower in hamsters exposed to either MSG or HFF diets. The reduction in the number of probiotics like *Lactobacillus* may have an impact on the level of uremic toxin. On the other hand, the proportion of harmful microorganisms, such as those belonging to the *Shigella* and *Escherichia* genus, grew in hamsters that were fed the HFF and/or MSG diet, as reported earlier (Kong et al., 2019). The *Firmicutes*/*Bacteroidetes* ratio indicates that the MSG + HFF diet intensifies gut dysbiosis in hamsters as a model. Thus, the dysbiosis initiates alteration of gut‐derived metabolites, including TMAO, indoxyl sulfate, and p‐cresol sulfate, which contribute to kidney injury (Hsu et al., 2018). The findings suggest that HFF and MSG intake resulted in an increase of p‐cresol sulfate levels, but a decrease in indoxyl sulfate and TMAO levels when compared to the control group. P‐cresol sulfate is identified as the primary component of urinary myelin's basic protein material, which can cause kidney tubular cell damage due to oxidative stress (Watanabe et al., 2013). The correlation of p‐cresol sulfate and *Akkermansia* has been recently reported (Visconti et al., 2019). HFF and the MSG diet in hamsters showed decreased TMAO levels in urine but increased *Methanobrevibacter* (MN326530) in the fecal samples. TMAO disrupts the changing of growth factor‐β (TGF‐beta)/Smad3 signaling pathway and impairs renal function (Sun et al., 2017). Recent studies have reported an association between the reduction of TMAO and *Methanobrevibacter* population (Ramezani et al., 2018); the reduction of TMAO might be due to the other gut microbiota activities. Nahok et al. (2021) have studied the effects of MSG intake on the gut microbiome and metabolic profiles of Wistar rats. The study found that rats treated with MSG showed changes in *Bacteroidetes* and *Firmicutes*. Specifically, the results showed a higher abundance of *Firmicutes* compared to *Bacteroidetes*. In addition, the study also revealed that the MSG‐treated mice had a higher abundance of *Clostridium* than *Bifidobacterium* and *Lactobacillus*. It is worth noting that the genus *Clostridium* includes the microorganisms that generally belong to the *Firmicutes* phylum, and are correlated with TMA metabolism by converting choline to TMA (Jameson et al., 2018; Rath et al., 2020). The increase of TMA‐producing bacterial species, that is, *Clostridium* spp., supports the increase of TMA metabolites in the urine and kidney of rats. Moreover, the MSG intake reduced the *Bifidobacterium* population, which plays a significant role in gut homeostasis and health (O'Callaghan & Van Sinderen, 2016). Previously, a reduction in *Bifidobacterium* abundance has been observed in hepatitis B and other chronic inflammatory diseases (Xu et al., 2012), diabetes (Murri et al., 2013), and obesity (Santacruz et al., 2010). The effect of MSG intake on the gut microbiota in humans was reported previously, and showed no important changes in the composition of gut microbiota compared to the baseline (Peng et al., 2018). Nonetheless, the low effect of MSG on the gut microbiota may be caused by the low dose supplementation such as 2 g/day, as the average intake of MSG is reported to be 4 g/day (Insawang et al., 2012). The *Bifidobacterium* population in MSG‐treated rats has been reduced which was comparable to rats with a lack of dietary vitamin B6 (Mayengbam et al., 2020), and further studies are needed to find how MSG decreases probiotic bacterial species and alters vitamin B6 levels.

Alongside these findings, most of the conducted studies are limited on animal models, especially rats, to verify if MSG is a risk factor of metabolic syndrome in humans, a longitudinal survey with a large sample size is required. This study must consider different nationalities, diets, and other confounding factors such as age, sex, physical activity, calorie intake, smoking status, and history of diabetes. Therefore, it is crucial to adjust the data accordingly to obtain accurate results. As metabolic syndrome is a growing global emergency, it is essential to investigate the potential risks associated with MSG consumption.

## 4 CONCLUSION

Excessive consumption of MSG can elevate the risk of cardiovascular diseases and disrupt the functioning of the gut microbiome. Furthermore, long‐term consumption of MSG can have significant effects on the metabolism of TMA, branched‐chain amino acids, and vitamin B6, as well as on the combined changes in the gut microbiota, renal, and hepatic metabolism. Evidence from studies on animal models demonstrated the association between MSG and the development of glucose intolerance, and obesity together with hypertrophy of adipose tissue, hyperglycemia, hyperinsulinemia, hyperleptinemia, and even decreased glucose transport in adipocytes and muscle. The outcomes of this study offer a new outlook and groundwork for future research on the impact and safety of MSG on human health.

## AUTHOR CONTRIBUTIONS

**Hossein Ahangari** and **Arezou Khezerlou**: Conceptualization, methodology, and software. **Hossein Ahangari** and **Behnam Bahramian**: Data curation and writing—original draft preparation. **Narges Kiani‐Salmi** and **Milad Tavassoli**: Visualization and investigation. **Ali Ehsani** and **Vahideh Tarhriz**: Supervision. **Hossein Ahangari** and **Behnam Bahramian**: Writing—reviewing and editing.

## FUNDING INFORMATION

This is a report of work registered in Tabriz University of Medical Sciences with the Number 70712.

## CONFLICT OF INTEREST STATEMENT

The authors declare that they do not have any conflict of interest.

## Supporting information


Table S1

## Data Availability

Data sharing is not applicable.
