# Correction to “Association Between Monosodium Glutamate Consumption with Changes in Gut Microbiota and Related Metabolic Dysbiosis—A Systematic Review”

**DOI:** 10.1002/fsn3.4584

**Published:** 2024-11-01

H. Ahangari, B. Bahramian, A. Khezerlou, M. Tavassoli, N. Kiani‐Salmi, V. Tarhriz, and A. Ehsani, “Association Between Monosodium Glutamate Consumption with Changes in Gut Microbiota and Related Metabolic Dysbiosis—A Systematic Review,” *Food Science & Nutrition* 12, no. 8 (2024): 5286–5295, https://doi.org/10.1002/fsn3.4198.

Figure 2 of the article was based on the graphical abstract of Nahok et al. (2021).

Please find the corrected Figure 2 caption below:

**FIGURE 2**: Mechanism of MSG and derived metabolites on gut microbiota modulations, kidney, and liver injury, adapted from the graphical abstract of Nahok et al. (2021).
