# Supplementary material for: Association between monosodium glutamate consumption with changes in gut microbiota and related metabolic dysbiosis—A systematic review
Source: Food Sci Nutr. 2024 Apr 29;12(8):5285–95. doi: 10.1002/fsn3.4198 (PMC11317663; doi:10.1002/fsn3.4198)
Supplement: Supplementary file 1 — Table S1 [file FSN3-12-5285-s001.docx]

**Supplementary Table 1.** Search strategies used for online databases

The following databases were searched:

- PubMed
- Scopus
- Web of Science

The search strategy was to combine searches of:

| Monosodium glutamate | "Monosodium glutamate" [MeSH Terms] OR "monosodium glutamate" [tiab] OR "flavor enhancer" [MeSH Terms] OR "flavor enhancer" [tiab] OR "umami" [MeSH Terms] OR "umami"[tiab] OR "mice" [MeSH Terms] OR "mice" [tiab] OR "rats" [MeSH Terms] OR "rats" [tiab] OR "toxicity" [MeSH Terms] OR "effects" [MeSH Terms] OR "obesity" [MeSH Terms] OR "obesity" [tiab] OR "hyperglycemia" [MeSH Terms] OR "hyperglycemia" [tiab] OR "metabolism" [MeSH Terms] OR "metabolism" [tiab] OR "liver toxicity" [MeSH Terms] OR "liver toxicity" [tiab] OR "nephrotoxicity" [MeSH Terms] OR "nephrotoxicity" [tiab] OR "oxidative stress" [MeSH Terms] OR "oxidative stress" [tiab] OR "health impact" [MeSH Terms] OR "health impact" [tiab] |
| --- | --- |
| Gut microbiota | "Gut microbiota" [MeSH Terms] OR "gut microbiota" [tiab] OR "gut flora" [MeSH Terms] OR "gut flora" [tiab] OR "microorganism" [MeSH Terms] OR "microorganism" [tiab] OR "Bacteroides" [MeSH Terms] OR "Bacteroides" [tiab] OR "Megamonas" [MeSH Terms] OR "Megamonas" [tiab] OR "Faecalibacterium" [MeSH Terms] OR "Faecalibacterium" [tiab] OR  "Blautia" [MeSH Terms] OR "Blautia" [tiab] OR "Collinsella" [MeSH Terms] OR "Collinsella" [tiab] |
| Study design | "Randomized"[Title/Abstract] OR "random"[Title/Abstract] OR "Intervention"[Title/Abstract] OR "Clinical trial"[Title/Abstract] OR "Randomized controlled trial"[Title/Abstract] OR "Randomized controlled trials"[Title/Abstract] OR "trial"[Title/Abstract] OR "Placebo"[Title/Abstract] OR "Double-blind"[Title/Abstract] OR "Single-blind"[Title/Abstract] OR "clinical trials as topic"[MeSH Terms] OR "random allocation"[MeSH Terms] OR "Randomised"[Title/Abstract] OR "Randomised clinical trials"[Title/Abstract] OR "Randomised clinical trial"[Title/Abstract] |

“Monosodium glutamate, “gut microbiota”, and “study design” related terms
